# Frequency and costs of low-value preoperative tests for patients undergoing low-risk procedures in the veterans health administration

**DOI:** 10.1186/s13741-022-00265-0

**Published:** 2022-09-13

**Authors:** Alex H. S. Harris, Thomas Bowe, Robin N. Kamal, Erika D. Sears, Mary Hawn, Dan Eisenberg, Andrea K. Finlay, Hildi J. Hagedorn, Seshadri Mudumbai

**Affiliations:** 1 Center for Innovation to Implementation, VA Palo Alto Healthcare System, Palo Alto, USA; 2 Stanford –Surgical Policy Improvement Research and Education Center, Department of Surgery, Stanford University School of Medicine, Palo Alto, USA; 3 Department of Orthopedic Surgery, Stanford University School of Medicine, Palo Alto, USA; 4 Center for Clinical Management Research, VA Ann Arbor Health Care System, Michigan Medicine Department of Surgery, Ann Arbor, USA; 5 Center for Care Delivery & Outcomes Research, Minneapolis Veterans Affairs Medical Center, Department of Psychiatry, University of Minnesota School of Medicine, Minneapolis, USA; 6 Department of Anesthesiology, Perioperative, and Pain Medicine, Stanford University School of Medicine, Palo Alto, USA

**Keywords:** Preoperative testing, Surgical care, Value, Efficiency, Implementation

## Abstract

**Background:**

Clinical practice guidelines discourage routine preoperative screening tests for patients undergoing low-risk procedures. This study sought to determine the frequency and costs of potentially low-value preoperative screening tests in Veterans Health Administration (VA) patients undergoing low-risk procedures.

**Methods:**

Using the VA Corporate Data Warehouse, we identified Operative Stress Score class 1 procedures (“very minor”) performed without general anesthesia in VA during fiscal year 2019 and calculated the overall national and facility-level rates and costs of nine common tests received in the 30 preoperative days. Patient factors associated with receiving at least one screening test, and the number of tests received, were examined.

**Results:**

Eighty-six thousand three hundred twenty-seven of 178,775 low-risk procedures (49.3%) were preceded by 321,917 potentially low-value screening tests representing $11,505,170 using Medicare average costs. Complete blood count was the most common (33.2% of procedures), followed by basic metabolic profile (32.0%), urinalysis (26.3%), electrocardiography (18.9%), and pulmonary function test (12.4%). Older age, female sex, Black race, and having more comorbidities were associated with higher odds of low-value testing. Transthoracic echocardiogram occurred prior to only 4.5% of the procedures but accounted for 47.8% of the total costs ($5,499,860). In 129 VA facilities, the facility-level proportion of procedures preceded by at least one test ranged from 0 to 81.2% and facility-level costs ranged from $0 to $388,476.

**Conclusions:**

Routine preoperative screening tests for very low-risk procedures are common and costly in some VA facilities. These results highlight a potential target to improve quality and value by reducing unnecessary care. Measures of low-value perioperative care could be integrated into VA’s extensive quality monitoring and improvement infrastructure.

**Supplementary Information:**

The online version contains supplementary material available at 10.1186/s13741-022-00265-0.

## Background

Screening tests prior to surgical and other medical procedures are justified if they produce actionable data that might alter clinical management or patient outcomes. However, for patients undergoing low-risk procedures, preoperative screening tests often do not change clinical management, sometimes lead to unnecessary follow-up testing and interventions, and can cause delays in receiving surgery (Schein et al. 2000; Lira et al. 2001; Cavallini et al. 2004; Keay et al. 2012; Saver 2015; Kirkham et al. 2016; Kirkham et al. 2015). To address this problem, the American Society of Anesthesiologists’ (ASA) Choosing Wisely Top-5 list of activities to avoid states: “Don’t obtain baseline laboratory studies in patients without significant systemic disease (ASA I or II) undergoing low-risk surgery - specifically complete blood count, basic or comprehensive metabolic panel, coagulation studies when blood loss (or fluid shifts) is/are expected to be minimal.” (Onuoha et al. 2014) National and international healthcare agencies have also issued similar guidance to avoid preoperative screening tests for low-risk procedures (Balk et al. 2014; National Institute for Health and Care Excellence 2016; Fleisher et al. 2015).

The existence of prominent guidance to avoid preoperative screening tests for low-risk procedures does not mean testing is never justified. For patients with infrequent contact with the healthcare system, surgical procedures can sometimes serve as opportunities to provide overdue screening and preventive care (Hambright et al. 2016; Wilson et al. 2012). For some patients with certain comorbidities or frailty, there may be anesthetic risks that justify testing even for low-risk procedures (Chassot et al. 2002). Thus, the recommendations to avoid preoperative testing for low-risk procedures should be interpreted to mean that testing should not be routine for all patients, not that testing should never be done. Nonetheless, except for these relatively rare situations, preoperative screening tests performed prior to minor surgery are often low value and should be avoided.

Even though guidance to avoid preoperative testing has been available for years, low-value preoperative tests continue to be a common and major contributor to unnecessary health care spending in the USA and Canada (Kirkham et al. 2016; Kirkham et al. 2015; Onuoha et al. 2015; Mafi et al. 2017; Schwartz et al. 2014). We previously found that 47.0% of generally healthy patients (ASA-PS I or II) undergoing a carpal tunnel release in the Veterans Health Administration (VA), the largest integrated healthcare system in the USA, had at least one low-value preoperative screening test within 30 days before surgery (Harris et al. 2019). We also found that 49% of 50,106 cataract surgeries performed in VA in 2017 were preceded by one or more preoperative screening test with an overall annual cost of $2.6 million (Mudumbai et al. 2021). In each of these studies, substantial variability existed between facilities, with some ordering almost zero tests and others routinely ordering tests for almost all patients undergoing these procedures. Although these studies highlight the need to better understand the drivers of low-value preoperative testing and what strategies might be used to improve practice, the data only involve two common procedures. It is unknown if these patterns of low-value testing generalize to a variety of other minor procedures.

To address this gap, this study had the following goals: (1) determine the overall and facility-level rates of receiving any of nine common low-value preoperative tests in the 30 days prior to any Operative Stress Score (OSS) I (“very minor”) procedures performed without general anesthesia in the VA in fiscal year 2019; (2) examine the patient factors that are associated with receiving at least one low-value test and the number of tests received; and (3) estimate the overall and facility-level costs of low-value preoperative testing. Knowing more about the overall burden of potentially unnecessary preoperative testing, as well as associated patient factors, might inform and motivate the development of interventions to reduce low-value care, especially in locations where the burden is highest.

## Methods

### Data source and cohort

All data were derived from the VA Corporate Data Warehouse (CDW), a nationwide database of all VA healthcare records. The cohort consisted of VA patients undergoing low-risk procedures in fiscal year 2019. More than one procedure per patient was included if the surgery dates were separated by at least 30 days.

### Defining low-risk procedures

The expanded Operative Stress Score (OSS) was developed to classify 5753 Category I Current Procedural Terminology (CPT) codes into five categories of physiologic stress (Yan et al. 2021; Shinall Jr et al. 2020). OSS 1-5 procedures are termed very low stress, low stress, moderate stress, high stress, and very high stress, respectively. The methodology of the expanded OSS and mapping of CPT codes to OSS categories are presented in the supplemental material of Yan et al. (Yan et al. 2021) OSS 1 includes 463 CPT codes for very low-stress procedures (e.g., wrist ganglion cyst excision, fasciotomy of foot and/or toe, carpal tunnel release). Due to the inherent risk of more sedating anesthesia that might justify use of screening tests, we excluded procedures performed with general anesthesia and included only those performed with monitored anesthesia care, spinal or regional block, or local anesthesia.

### Defining preoperative screening tests

Preoperative tests were identified using CPT codes recorded in the CDW in the 30 days before the OSS1 procedure. Tests included complete blood count (CBC); basic metabolic panel (BMP); coagulation tests; urinalysis; electrocardiography (EKG); pulmonary function tests (PFT); trans-thoracic echocardiograms (TTE); cardiac stress tests, and chest x-rays. Tests were excluded if they occurred within 30 days prior to an OSS 2–5 procedure that may have justified it.

### Patient characteristics

Patient characteristics included age at time of the procedure, gender, race/ethnicity, marital status, service connection status (a measure of socio-economic status and medical need tied to military service), and 30 diagnoses included in the Elixhauser Comorbidity Index (Southern et al. 2004) recorded in the preoperative year.

### Estimating costs

To provide an approximation of the financial impact of testing, Centers for Medicare Services (CMS) reimbursement Fee Schedule for physician fees, facility fees, and the CMS clinical laboratory fee schedule for 2019 were used to assign a cost to each preoperative screening test.

### Statistical analysis

Overall and facility-level rates of OSS 1 procedures that were preceded by at least one low-value test were calculated. Rates of OSS 1 procedures preceded by each of the nine tests were also calculated. Mixed-effects logistic regression was used to examine associations between patient characteristics (e.g., demographics, comorbidities) and receipt of at least one low-value test, with random intercepts for the VA facility where the procedure was performed (Pinheiro and Bates 2000). Mixed-effects negative binomial regression was used to examine associations between the same patient, procedure, and facility characteristics and the number of low-value tests received. Odds ratios, 95% confidence intervals, and *p* values were produced for all regression model coefficients.

## Results

In fiscal year 2019, 148,728 VA patients received 178,775 OSS I procedures without general anesthesia, of which 86,327 (49.3%) involved at least one screening test in the 30 preoperative days. Among the 44,545 OSS I procedures that were excluded involving general anesthesia, 82.5% were preceded by at least one screening test. Characteristics of the cohort, stratified by receipt of any preoperative screening test, are presented in Table 1.

**Table 1:** Characteristics of Veterans Health Administration patient receiving OSS 1 procedures without general anesthesia in fiscal year 2019

Patient characteristics (%)	Any preop-test		
	No 92,448 (51.7)	Yes 86,327 (48.3)	Total 178,775^a^
Age, mean (*SD*)	65.5 (15.0)	68.3 (12.3)	66.9 (13.8)
Gender, No. (%)			
Male	83,029 (50.9)	80,236 (49.1)	163,265 (91.3)
Female	9419 (60.7)	6091 (39.3)	15,510 (8.7)
Race/ethnicity, No. (%)			
Native American	676 (53.9)	579 (46.1)	1254 (0.7)
Asian	663 58.0)	480 (42.0)	1143 (0.6)
Black	16,371 (49.4)	16,789 (50.6)	33,160 (18.5)
Hawaiian, Pacific Islander	724 (50.8)	700 (49.2)	1424 (0.8)
White	69,240 (52.1)	63,662 (47.9)	132,902 (74.3)
Missing	4774 (53.7)	4118 (46.3)	8892 (5.0)
Marital status, No. (%)			
Married	47,703 (51.9)	44,263 (48.1)	91,966 (51.4)
Not married	44,745 (51.5)	42,064 (48.5)	86,806 (48.6)
Service connection			
Less than 50%	52,707 (51.6)	49,358 (48.4)	102,065 (57.1)
More than 50%	39,741 (51.8)	36,969 (48.2)	76,710 (42.9)
Past year elixhauser comorbidities, No. (%)			
Congestive heart failure	6732 (31.4)	14,728 (68.6)	21,460 (12.0)
AIDS	495 (39.1)	772 (60.9)	1267 (0.7)
Cardiac arrhythmia	13,972 (37.4)	23,340 (62.6)	37,312 (20.9)
Pulmonary circulation disorders	1631 (28.1)	4183 (71.9)	5814 (3.2)
Peripheral vascular disease	10,434 (39.2)	16,197 (60.8)	26,631 (14.9)
Hypertension	49,644 (46.2)	57,685 (53.7)	107,329 (60.0)
Hypertension with complications	8221 (31.4)	17,926 (68.6)	26,147 (14.6)
Paralysis	1587 (42.8)	2123 (57.2)	3710 (2.1)
Other neurological disorders	5821 (40.0)	8766 (60.0)	14,587 (8.2)
Chronic pulmonary disease	17243 (41.1)	24,665 (28.9)	41,908 (23.4)
Diabetes mellitus	22,651 (43.8)	29,070 (56.2)	51,721 (28.9)
Diabetes mellitus with complications	17,726 (40.5)	26,055 (59.5)	43,781 (24.5)
Hypothyroidism	6928 (44.4)	8664 (55.6)	15,592 (8.7)
Renal failure	10,285 (33.5)	20,390 (66.5)	30,675 (17.2)
Liver disease	5815 (38.0)	9479 (62.0)	15,294 (8.6)
Peptic ulcer	502 (32.1)	1064 (67.9)	1566 (0.9)
Lymphoma	748 (24.5)	2309 (75.5)	3057 (1.7)
Metastatic cancer	1506 (21.9)	5379 (78.1)	6885 (3.9)
Solid tumor w/o metastasis	25,998 (47.7)	28,483 (52.3)	54,481 (30.5)
Rheumatoid arthritis	2191 (42.9)	2912 (57.1)	5103 (2.9)
Coagulopathy	1950 (27.9)	5040 (72.1)	6990 (3.9)
Obesity	15,790 (46.2)	18,363 (53.8)	34,153 (19.1)
Weight loss	2912 (29.6)	6932 (70.4)	9844 (5.5)
Fluid and electrolyte disorders	6378 (26.8)	17,383 (73.2)	23,761 (13.3)
Blood loss anemia	731 (25.9)	2087 (74.1)	2818 (1.6)
Deficiency anemia	4904 (32.9)	9998 (67.1)	14,902 (8.2)
Alcohol abuse	2257 (35.4)	4114 (64.6)	6371 (3.6)
Drug abuse	4445 (45.8)	5253 (54.1)	9698 (5.4)
Psychosis	1602 (44.0)	2040 (56.0)	3642 (2.0)
Depression	22,404 (48.0)	24,295 (52.0)	46,699 (26.1)

^a^Total represents 178,775 procedures (separated by at least 30 days) in 148,728 patients.

As presented in Table 2, complete blood count was the most common (33.2% of procedures), followed by basic metabolic profile (32.0%), urinalysis (26.3%), electrocardiogram (18.9%), and pulmonary function test (12.4%). The least common screening tests were cardiac stress tests (2.0% of procedures), coagulation tests (<1.0%), and chest x-rays (0.0%). Overall, we identified 321,917 preoperative screening tests in the 30 days prior to the 178,775 OSS 1 procedures, representing $11,505,170 in Medicare Average Costs. Transthoracic echocardiogram occurred prior to only 4.5% of the procedures but accounted for 47.8% of the total costs ($5,499,860).

**Table 2:** Low-value preoperative testing prior to 178,775 OSS 1 procedures in Veterans Health Administration during fiscal year 2019

Preoperative test	Percent received >=1 tests	Total number	Total cost
Complete blood count	33.2%	77,497	$805,438
Basic metabolic profile	32.0%	70,240	$912,317
Urinalysis	26.3%	55,122	$200,930
Electrocardiography	18.9%	54,310	$742,460
Pulmonary function tests	12.4%	52,311	$852,580
Transthoracic echocardiogram	4.5%	8424	$5,499,860
Cardiac stress test	2.0%	3759	$2,490,125
Coagulation test	<1%	254	$1458
Chest x-ray	0%	0	0
Total	48.3%	321,917	$11,505,170

**Fig. 1:**
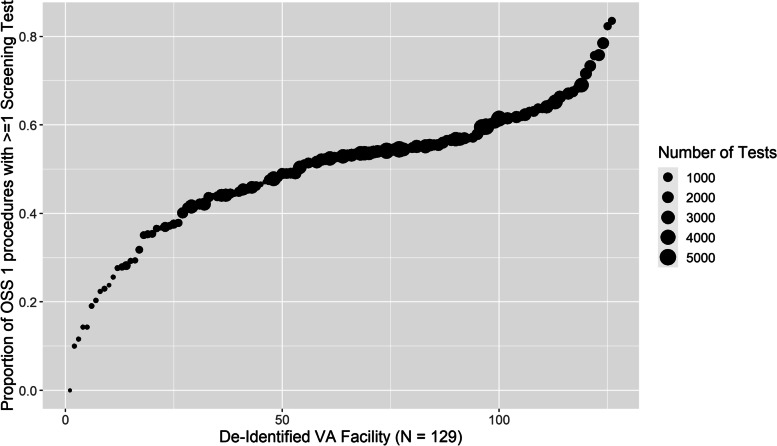
The proportion of OSS 1 procedures proceeded by at least 1 low-value test in 129 VA facilities

**Fig. 2:**
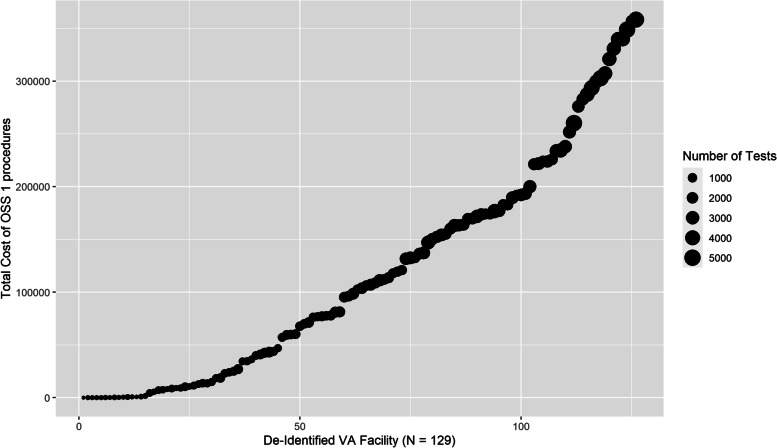
Total cost low-value screening test prior to OSS 1 procedures in 129 VA facilities

In 129 VA facilities, the facility-level proportion of low-risk procedures preceded by at least one test ranged from 0 to 81.2% (Fig. 1), and facility-level total costs ranged from $0 to $388,476 with a median total cost of $69,786 (Fig. 2). The top quartile of facilities with the highest testing cost accounted for 57% of total costs. In mixed effect logistic regression (Table 3), older age, being female, not married, Black, or having service-connected status greater than 50% were associated with higher odds of preoperative testing. Every comorbidity in the Elixhauser Comorbidity Index except peptic ulcer was associated with higher odds of testing. The intraclass correlation coefficient (ICC)—the proportion of outcome variance attributable to facility rather than patient factors—was 0.189. The beta-binomial regression model examining factors associated with the number of preoperative tests received found the same pattern of results (Supplemental Table 1) and had an ICC = 0.02.

**Table 3:** Patient characteristics associated with odds of receiving at least one preoperative screening test

Factor	OR	LL	UL	*p* value
Intercept	0.43	0.33	0.53	0.00
Age	1.01	1.00	1.01	0.00
Female	1.06	1.02	1.10	0.00
Not married	1.03	1.01	1.05	0.01
Service connected >50%	1.02	1.00	1.04	0.05
Native American (ref = white)	1.05	0.93	1.17	0.40
Asian	1.01	0.88	1.13	0.92
Black	1.04	1.01	1.07	0.00
Hawaiian	1.07	0.95	1.18	0.26
Race missing	0.98	0.93	1.03	0.44
Peptic ulcer	1.08	0.96	1.20	0.19
AIDS	1.42	1.30	1.54	0.00
Lymphoma	2.66	2.57	2.75	0.00
Metastatic cancer	3.42	3.36	3.48	0.00
Solid tumor no metastasis	0.94	0.91	0.96	0.00
Rheumatoid arthritis	1.25	1.19	1.31	0.00
Coagulopathy	1.42	1.36	1.48	0.00
Obesity	1.09	1.06	1.11	0.00
Weight loss	1.41	1.36	1.46	0.00
Fluid and electrolyte disorders	1.69	1.66	1.73	0.00
Blood loss anemia	1.39	1.29	1.48	0.00
Deficiency anemia	1.19	1.14	1.23	0.00
Alcohol abuse	1.12	1.06	1.18	0.00
Drug abuse	1.06	1.02	1.11	0.01
Psychosis	1.10	1.02	1.17	0.01
Depression	1.06	1.04	1.09	0.00
Congestive heart failure	1.22	1.18	1.26	0.00
Cardiac arrhythmia	1.32	1.29	1.34	0.00
Valvular disorder	1.23	1.18	1.28	0.00
Pulmonary circulation disorder	1.24	1.17	1.30	0.00
Peripheral vascular disorder	1.07	1.04	1.10	0.00
Hypertension	1.18	1.16	1.21	0.00
Hypertension with complications	1.12	1.08	1.15	0.00
Paralysis	1.19	1.12	1.27	0.00
Other neurological disorder	1.09	1.05	1.13	0.00
Chronic pulmonary disease	1.27	1.24	1.29	0.00
Diabetes melitus	1.07	1.04	1.10	0.00
Diabetes melitus with complications	1.14	1.11	1.17	0.00
Hypothyroidism	1.06	1.02	1.09	0.00
Renal failure	1.37	1.33	1.40	0.00
Liver disease	1.33	1.29	1.37	0.00

## Discussion

High-value, patient-centered health care includes selectively ordering preoperative tests that may inform clinical management or improve patient outcomes. Tests unlikely to meet these criteria may cause avoidable harm, inconvenience, and waste of resources that could be used for higher-value services. For years, government healthcare agencies and professional organizations have been recommending the avoidance of routine preoperative testing for low-risk procedures (Balk et al. 2014; National Institute for Health and Care Excellence 2016; Fleisher et al. 2015). However, we found that almost half of OSS 1 procedures received by VA patients in FY19 were preceded by at least one potentially low-value preoperative test. Using Medicare Average Costs, we estimated that the 321,917 preoperative screening tests prior to OSS 1 procedures may represent up to $11,505,170 in low-value care.

As noted, some portion of this testing may represent high-quality care (e.g., opportunities for overdue screening) or tests that are unrelated to the OSS1 procedures. If the distribution of testing was uniformly modest (e.g., 5 or 10%), the case for investments in quality improvement might be easier to dismiss. However, we can see in Figs. 1 and 2 that there exist many VA facilities that routinely test the vast majority of patients prior to low-risk procedures, and 27 facilities with estimated associated costs over $200,000. For these facilities, these results highlight a significant opportunity to improve quality by providing less unnecessary care.

Beyond describing the magnitude and distribution of opportunities for quality improvement, another purpose of this study was to identify the patient characteristics associated with preoperative testing. We found older age, being female, not married, black, and having comorbidities were all associated with higher odds of testing. The large ICC in the model predicting receipt of at least one test (0.189) suggests that much of the variance in preoperative testing is at the facility-level. In other words, the likelihood of getting tested is as much a function of where you are treated as your specific medical profile.

Although excluded from our primary analyses, general anesthesia was received for 16.5% of OSS1 procedures in FY19, of which 82.2% underwent preoperative testing. Anesthetic risk, not just procedural risk, needs to be factored into the decision to order preoperative tests. We previously found that much of the variance in using general anesthesia for a low-risk procedure (carpal tunnel release) is driven by clinician or facility factors rather than patient characteristics or preferences (Harris et al. 2020). Therefore, although preoperative testing may be justified for patients undergoing general anesthesia, there may be quality improvement opportunities in facilities that commonly or routinely use general anesthesia even for low-risk procedures.

Several limitations are worth noting. First, there is no way to be sure that tests in the 30 days prior to OSS1 procedures were ordered for preoperative screening purposes. Some of the tests we identified may be justified by factors independent of the upcoming low-risk procedure. However, we have no reason to expect that such justifications for the tests differ systematically between facilities. In our previous work on cataract and carpal tunnel release surgery, we excluded 10% of preoperative tests because they were not preceded by a ‘plausible ordering visit, such as ophthalmology or anesthesia consult. As we could not implement this methodology in this study due to the diversity of procedures, it is possible that our estimates of low-value testing are 10% too high due to this limitation. Also, it is unknown to what extent these results might generalize outside of the VA system.

## Conclusions

In summary, low-value preoperative screening tests for patients undergoing low-risk procedures without general anesthesia appear to be common and costly in the VA system, with the burden of this low-value care concentrated in certain facilities. One obvious way to begin to address the burden of low-value preoperative testing is to develop quality measures of low-value perioperative care that could be integrated into VA’s extensive quality monitoring and improvement infrastructure. By identifying facilities with the highest burden of low-value care, then seeking to identify its root causes, interventions (e.g., educational, informatics, behavioral) can be designed and implemented to improve the quality of care by providing less of it.

## Supplementary Information

**Additional file 1: Supplemental Table 1.** Patient Characteristics Associated with Number of Preoperative Screening Tests Received.

## Data Availability

VA patient data are not publicly. Please contact author for aggregate data requests.

## Abbreviations

VA: Veterans Health Administration
ASA: American Society of Anesthesiologists
OSS: Operative Stress Score
CDW: Corporate Data Warehouse
CPT: Current Procedural Terminology
CBC: Complete blood count
BMP: Basic metabolic panel
EKG: Electrocardiography
PFT: Pulmonary function tests
TTE: Trans-thoracic echocardiograms
CMS: Centers for Medicare Services
ICC: Intraclass correlation coefficient
FY: Fiscal year
