# Supplementary material for: Frequency and costs of low-value preoperative tests for patients undergoing low-risk procedures in the veterans health administration
Source: Perioper Med (Lond). 2022 Sep 13;11:33. doi: 10.1186/s13741-022-00265-0 (PMC9469517; doi:10.1186/s13741-022-00265-0)
Supplement: Supplementary file 1 — Additional file 1: Supplemental Table 1. Patient Characteristics Associated with Number of Preoperative Screening Tests Received. [file 13741_2022_265_MOESM1_ESM.docx]

Supplemental Table 1: Patient Characteristics Associated with Number of Preoperative Screening Tests Received

| Factor | OR | LL | UL | p-value |
| --- | --- | --- | --- | --- |
| Intercept | 0.76 | 0.69 | 0.82 | 0.00 |
| Age | 1.00 | 1.00 | 1.00 | 0.43 |
| Female | 1.14 | 1.11 | 1.17 | 0.00 |
| Not Married | 1.04 | 1.02 | 1.05 | 0.00 |
| Service Connected >50% | 0.98 | 0.97 | 1.00 | 0.03 |
| Native American (ref – white) | 0.94 | 0.84 | 1.04 | 0.24 |
| Asian | 1.03 | 0.93 | 1.13 | 0.58 |
| Black | 1.02 | 1.00 | 1.04 | 0.03 |
| Hawaiian | 0.97 | 0.89 | 1.06 | 0.50 |
| Race Missing | 1.06 | 1.02 | 1.09 | 0.00 |
| Peptic ulcer | 0.99 | 0.94 | 1.05 | 0.82 |
| AIDS | 1.09 | 1.01 | 1.16 | 0.03 |
| Lymphoma | 1.58 | 1.54 | 1.62 | 0.00 |
| Metastatic Cancer | 1.61 | 1.57 | 1.64 | 0.00 |
| Solid tumor no metastasis | 0.86 | 0.84 | 0.88 | 0.00 |
| Rheumatoid Arthritis | 1.18 | 1.15 | 1.22 | 0.00 |
| Coagulopathy | 1.21 | 1.19 | 1.24 | 0.00 |
| Obesity | 1.02 | 1.00 | 1.04 | 0.01 |
| Weight loss | 1.37 | 1.34 | 1.39 | 0.00 |
| Fluid and electrolyte disorders | 1.66 | 1.64 | 1.68 | 0.00 |
| Blood loss anemia | 1.15 | 1.11 | 1.19 | 0.00 |
| Deficiency anemia | 1.05 | 1.03 | 1.08 | 0.00 |
| Alcohol abuse | 1.03 | 1.00 | 1.07 | 0.06 |
| Drug abuse | 1.03 | 1.00 | 1.06 | 0.04 |
| Psychosis | 1.19 | 1.14 | 1.23 | 0.00 |
| Depression | 1.03 | 1.01 | 1.05 | 0.00 |
| Congestive heart failure | 1.24 | 1.22 | 1.26 | 0.00 |
| Cardiac Arrhythmia | 1.37 | 1.35 | 1.39 | 0.00 |
| Valvular disorder | 1.08 | 1.06 | 1.11 | 0.00 |
| Pulmonary circulation disorder | 1.26 | 1.24 | 1.29 | 0.00 |
| Peripheral vascular disorder | 0.95 | 0.93 | 0.97 | 0.00 |
| Hypertension | 1.09 | 1.07 | 1.11 | 0.00 |
| Hypertension with complications | 0.98 | 0.96 | 1.01 | 0.18 |
| Paralysis | 1.71 | 1.67 | 1.75 | 0.00 |
| Other neurological disorder | 1.05 | 1.03 | 1.08 | 0.00 |
| Chronic pulmonary disease | 1.45 | 1.43 | 1.46 | 0.00 |
| Diabetes Melitus | 1.00 | 0.98 | 1.02 | 0.81 |
| Diabetes Melitus with complications | 1.07 | 1.05 | 1.10 | 0.00 |
| Hypothyroidism | 1.04 | 1.01 | 1.06 | 0.00 |
| Renal failure | 1.36 | 1.34 | 1.38 | 0.00 |
| Liver disease | 1.13 | 1.11 | 1.15 | 0.00 |
